# Effect of Biotic and Abiotic Factors on the Physiology of Horticultural Plants

**DOI:** 10.3390/plants15060961

**Published:** 2026-03-20

**Authors:** Filippos Bantis, George Zervoudakis

**Affiliations:** 1 Department of Agriculture, University of Western Macedonia, 53100 Florina, Greece; 2 Department of Agriculture, University of Patras, 30200 Messolonghi, Greece

Horticultural crops comprise a diverse group of intensively cultivated plant species including vegetables, fruits, ornamental plants, and medicinal or aromatic crops, which are typically characterized by high economic value, intensive management, and strong dependence on environmental conditions. These crops play a crucial role in global food production and economic sustainability. However, their productivity and quality are increasingly challenged by environmental pressures, including climate crisis, abiotic constraints such as salinity, extreme temperatures, and water deficit, as well as biotic stresses. Understanding how plants respond to these factors is essential for developing resilient production systems. The Special Issue “Effect of Biotic and Abiotic Factors on the Physiology of Horticultural Plants” was organized to advance knowledge on both plant physiological responses and stress tolerance mechanisms and innovative management strategies.

Another important challenge facing modern horticulture is the genetic narrowing that has resulted from intensive breeding programs primarily targeting high yield, uniformity, and market-related traits. While these efforts have significantly improved productivity and product quality, they have often been accompanied by the loss or underutilization of genetic traits associated with tolerance to environmental stresses. As climate variability intensifies, the reintegration of such traits has become increasingly important for restoring resilience in elite cultivars. These traits are frequently present in landraces, wild relatives, and less intensively bred genotypes. Understanding the physiological basis of plant responses to biotic and abiotic stresses is therefore essential for guiding breeding strategies aimed at improving crop adaptability under changing environmental conditions.

The contributions gathered in this Special Issue explore the diverse interactions between horticultural plants and their environments. Several studies focus on responses to abiotic parameters, highlighting how factors such as salinity [1,2,3,4], drought [5], and root-zone temperature [6] induce oxidative damage and metabolic alterations, while also demonstrating the potential of biostimulants or adaptive mechanisms to mitigate stress effects and improve plant performance. Other contributions [7,8] investigate plant–pathogen interactions and defense mechanisms, revealing the complex roles of antioxidant systems, phytohormone signaling, and physiological adjustments in plant resistance against diseases. Another contribution [9] deals with the phenotypic plasticity and adaptive strategies of riparian plants in heterogeneous light habitats.

Soil salinity is a major limitation for strawberry cultivation worldwide, compromising vegetative growth, fruit yield, and marketable quality. Developing integrated strategies that combine physiological understanding with biological interventions and breeding approaches is therefore essential for sustaining production in salt-affected regions. In the review of Passa et al. [1], the authors synthesize current knowledge on salinity-induced physiological, biochemical, and molecular responses in strawberry, while critically evaluating the role of biostimulants, signaling molecules, beneficial microorganisms, and emerging genetic tools in enhancing salt tolerance. They highlight the complexity of osmotic adjustment, antioxidant defense, ion homeostasis, and stress-responsive gene networks underlying plant adaptation. Importantly, the review emphasizes that many identified tolerance mechanisms are characterized at early developmental stages and do not always translate into stable improvements in fruit yield and quality. Significant genotypic variation among cultivars and crop wild relatives is discussed as a valuable resource for breeding, although stress-response traits must be carefully validated for agronomic relevance. Overall, the work underscores the need to bridge mechanistic knowledge with long-term field performance to develop truly salt-resilient strawberry cultivars.

Salinity and alkaline stress also severely constrain maize productivity worldwide, primarily through oxidative damage and impairment of photosynthesis. Identifying biostimulant-based strategies to reinforce physiological resilience under such conditions is therefore of major agronomic importance. In the study of Qi et al. [2], the authors investigated the role of exogenous melatonin (150 μM) in mitigating 100 mM Na_2_CO_3_ stress in a salt-susceptible maize line (P138) and its salt-resistant EMS mutant, combining physiological measurements with qRT-PCR analysis of antioxidant- and pigment-related genes. They demonstrated that melatonin application improved relative water content, chlorophyll concentration, and gas exchange parameters (Pn, Gs, Tr), while reducing membrane injury and H_2_O_2_ accumulation. Moreover, key genes associated with antioxidant defense and photosynthetic pigment biosynthesis were upregulated, indicating coordinated transcriptional regulation. Overall, their findings show that melatonin preserves photosynthetic apparatus integrity, enhances ROS scavenging, and strengthens maize seedling tolerance to alkaline salt stress.

Mild salinity can act not only as a stress factor but also as a regulatory signal influencing early plant development, making its precise management highly relevant for seedling production systems. Understanding how low salt concentrations modulate water transport and growth processes is particularly important for controlled environments such as hydroponics and greenhouses. Masepan et al. [3] investigated the effects of low NaCl concentrations (10 and 20 mM) on Chinese white radish seedlings grown in sand culture, evaluating growth traits, ion accumulation, water status, aquaporin gene expression, and physiological parameters four days after sowing. They demonstrated that 10 mM NaCl markedly stimulated cotyledon growth, increasing biomass, area, thickness, and mesophyll cell size. This enhancement was associated with elevated Na^+^, Cl^−^, K^+^, nitrogen, carbon, and proline contents, as well as upregulation of key aquaporin genes (PIP and TIP isoforms), indicating improved water transport capacity. Additionally, maintenance of Hill reaction activity and enhanced antioxidant capacity supported greater cell expansion and succulence. Collectively, the findings suggest that low NaCl application can function as a simple agronomic strategy to promote early seedling vigor through aquaporin-mediated water regulation.

Salinity is also a major constraint in coastal and marginal agricultural systems, affecting both plant productivity and the biosynthesis of valuable secondary metabolites, even in halophytic species. Enhancing salt tolerance while preserving phytochemical quality is therefore essential for the sustainable cultivation of medicinal and aromatic crops such as *Crithmum maritimum*. In the study of Giannakoula et al. [4], the authors evaluated the physiological, antioxidant, and essential oil responses of sea fennel under increasing NaCl salinity (10 and 20 dS m^−1^) combined with foliar application of two biostimulants, Aquamin and Cultisano, assessing growth traits, oxidative stress markers, photosynthetic performance, and volatile compound profiles (GC–MS). They found that moderate salinity stimulated growth and photosynthesis, whereas high salinity reduced biomass, photosynthetic efficiency, and water use efficiency, accompanied by elevated MDA and H_2_O_2_ levels. Biostimulant treatments mitigated oxidative damage, improved pigment retention and antioxidant capacity, and partially restored photosynthetic activity under severe salinity. Moreover, they modulated essential oil composition, with Aquamin particularly enhancing p-cymene and stabilizing monoterpenes, while Cultisano promoted specific compounds such as thymol-methyl-ether. Overall, the study demonstrates that targeted biostimulant applications can simultaneously enhance salt resilience and phytochemical value in halophytic crops, supporting their sustainable exploitation in saline environments.

Drought stress is among the most critical constraints to vegetable production, particularly for pepper, a crop highly sensitive to water deficits due to its physiological characteristics. Identifying genotypes that combine yield stability with fruit quality under limited irrigation is therefore essential for sustainable production and breeding programs. Ntanasi et al. [5] evaluated the effects of a 40% reduction in irrigation on yield, macronutrient composition, and fruit quality traits in two landraces (‘JO109’, ‘JO204’), the cultivar ‘Yolo Wonder’, and the commercial hybrid ‘Sammy RZ’, under greenhouse conditions. They found that most cultivars suffered significant yield reductions due to decreased fruit number or weight; however, the landrace ‘JO109’ maintained stable yields under drought stress. This resilience was associated with higher leaf K concentrations and lower Na accumulation in fruits, underlining the protective role of potassium in stress mitigation. Moreover, deficit irrigation enhanced fruit firmness and total soluble solids, particularly in ‘JO109’, without negatively affecting titratable acidity, indicating that certain genotypes can preserve both productivity and quality under water-limited conditions.

Root-zone temperature is a decisive factor in soilless cultivation systems, directly influencing root activity, nutrient uptake, and ultimately crop productivity and quality. Optimizing this parameter is particularly important for leafy vegetables grown in NFT and aeroponic systems during cool periods. Bantis et al. [6] evaluated mini Romaine lettuce cultivated in nutrient film technique and aeroponics under heated nutrient solutions (14, 18, and 22 °C) compared with ambient conditions (11–12 °C), assessing yield, root growth, photosynthetic performance, pigment content, and nutritional traits. They demonstrated that increasing root-zone temperature, particularly to 22 °C, substantially enhanced leaf biomass and leaf length in both systems, with more pronounced effects in NFT. Suboptimal temperatures limited root development and biomass accumulation despite relatively maintained photosynthetic activity, indicating sink-related growth restrictions. In NFT, moderate heating also reduced nitrate accumulation and increased total soluble solids, while pigment responses varied between systems. Overall, the findings highlight that targeted root-zone heating can significantly improve production efficiency in soilless lettuce cultivation, especially in low- and mid-tech greenhouses during cooler months.

Gray mold, caused by *Botrytis cinerea*, is one of the most destructive diseases in grape production worldwide, posing major challenges for yield and fruit quality. Understanding the molecular interplay between secondary metabolism and plant defense responses is therefore crucial for developing more resistant cultivars. In the study of Hao et al. [7], the authors investigated the role of VvMYBPA1, a key transcription factor regulating proanthocyanidin biosynthesis, using agroinfiltration-mediated transient overexpression in grape berries and subsequent analysis of antioxidant enzymes and ROS-related genes. They found that overexpression of VvMYBPA1 increased susceptibility to *B. cinerea*, despite enhancing proanthocyanidin accumulation. This heightened susceptibility was associated with reduced β-1,3-glucanase and polyphenol oxidase activities, increased expression of respiratory burst oxidase homologs (*VvRBOHs*), decreased peroxidase activity, and excessive H_2_O_2_ accumulation leading to enhanced cell death. Overall, the study demonstrates that VvMYBPA1 negatively regulates grape resistance to gray mold by disturbing ROS homeostasis, providing valuable knowledge for future genome-editing strategies targeting MYB transcription factors to improve disease resistance.

Leaf blight represents a serious threat to ornamental crops, compromising both aesthetic quality and commercial value, particularly in high-value shrubs such as *Ilex verticillata*. A deeper understanding of pathogen identity and host defense mechanisms is essential for developing effective disease management strategies. In this study, Lu et al. [8] identified *Alternaria alternata* as the causal agent of leaf blight through morphological characterization, multi-locus molecular analyses (ITS, TEF1-α, G3PDH, RPB2), and pathogenicity assays fulfilling Koch’s postulates, and further explored host responses using transcriptomic, physiological, and phytohormone analyses. They revealed a dynamic, multi-layered defense response characterized by early activation of pattern-recognition receptors and antioxidant enzymes, followed by extensive metabolic reprogramming. An early oxidative burst, indicated by increased malondialdehyde levels, was accompanied by sustained antioxidant activity (SOD, CAT), reflecting adaptive stress mitigation. Moreover, distinct salicylic acid and jasmonic acid crosstalk, with a mid-infection SA peak and a later JA rebound, highlighted coordinated hormonal regulation of defense. Collectively, these findings elucidate the complex antioxidant and phytohormone-mediated responses of *I. verticillata* to *A. alternata*, offering valuable targets for breeding and hormone-based disease management strategies.

Understanding how riparian plants adjust their structural and physiological traits to heterogeneous light environments is essential for interpreting ecological adaptation and energy allocation strategies. In dynamic riverine systems, coordinated variation between twig and leaf traits, together with thermal dissipation capacity, plays a key role in maintaining photosynthetic performance. Li et al. [9] examined populations of *Hippophae rhamnoides* along three canopy light gradients (full light, moderate shade, and canopy cover) in the Taohe River riparian zone, assessing twig–leaf morphological traits and chlorophyll fluorescence parameters to explore their relationship with leaf thermal dissipation. They found significant shifts in twig architecture, specific leaf area (SLA), leaf thickness, and energy partitioning across habitats. In high-light environments, plants developed thicker leaves with lower SLA and allocated a greater proportion of absorbed light energy to heat dissipation, enhancing photoprotection. Under shaded and canopy-covered conditions, morphological adjustments and reduced thermal dissipation reflected strategies to optimize light capture, balance competition, and maintain photosynthetic stability. Overall, the coordinated variation in twig–leaf traits and fluorescence characteristics highlights the phenotypic plasticity and adaptive strategies of riparian plants in heterogeneous light habitats.

This Special Issue underscores the relevance of plant physiology research for sustainable horticulture. Advances in stress physiology can guide the development of cultivation practices, breeding strategies, and innovative inputs aimed at improving crop resilience to produce more with fewer resources. In conclusion, the studies included in this Special Issue contribute to a deeper understanding of how biotic and abiotic factors affect horticultural plant physiology. By bridging fundamental research with practical applications, this collection highlights emerging directions in stress biology and sustainable crop production, offering valuable perspectives for researchers, agronomists, and practitioners working toward resilient horticultural systems.
